# Imaging-guided chest biopsies: techniques and clinical results

**DOI:** 10.1007/s13244-017-0561-6

**Published:** 2017-06-21

**Authors:** Michele Anzidei, Andrea Porfiri, Fabrizio Andrani, Michele Di Martino, Luca Saba, Carlo Catalano, Mario Bezzi

**Affiliations:** 1 Department of Radiological, Oncological and Anatomopathological Sciences, Radiology, Sapienza, University of Rome, Policlinico Umberto I, Viale Regina Elena, 324, 00161 Rome, Italy; 2 Department of Radiology, Azienda Ospedaliero Universitaria (A.O.U.), di Cagliari, Polo di Monserrato Italy

**Keywords:** Lung lesion, Percutaneous biopsy, Complication, Diagnostic accuracy, Fine-needle aspiration biopsy (FNAB), Core-needle biopsy (CNB)

## Abstract

**Background:**

This article aims to comprehensively describe indications, contraindications, technical aspects, diagnostic accuracy and complications of percutaneous lung biopsy.

**Methods:**

Imaging-guided biopsy currently represents one of the predominant methods for obtaining tissue specimens in patients with lung nodules; in many cases treatment protocols are based on histological information; thus, biopsy is frequently performed, when technically feasible, or in case other techniques (such as bronchoscopy with lavage) are inconclusive.

**Results:**

Although a coaxial system is suitable in any case, two categories of needles can be used: fine-needle aspiration biopsy (FNAB) and core-needle biopsy (CNB), with the latter demonstrated to have a slightly higher overall sensitivity, specificity and accuracy.

**Conclusion:**

Percutaneous lung biopsy is a safe procedure even though a few complications are possible: pneumothorax, pulmonary haemorrhage and haemoptysis are common complications, while air embolism and seeding are rare, but potentially fatal complications.

***Teaching points*:**

*• Imaging-guided biopsy is one of the main methods to obtain lung nodule specimens*.

*• CT has the highest accuracy for diagnosis as an imaging guide*.

*• Compared to FNAB, CNB has a higher accuracy for diagnosis*.

*• Pneumothorax and parenchymal pulmonary haemorrhage care the most frequent complications*.

*• Several clinical and technical variables can affect diagnostic accuracy and patient safety*.

## Introduction

Chest tumours, in particular lung cancer, remain one of the most common causes of cancer-related death worldwide. With the diffusion of spiral CT, an increasing number of lung and mediastinal lesions is detected and histological diagnosis is often necessary to determine the most appropriate management of these lesions [1]. In this clinical scenario, imaging-guided biopsy is one of the main methods to obtain tissue specimens. Various imaging techniques including computed tomography (CT) fluoroscopy and ultrasound (US) can be used to guide chest biopsies, but CT is most frequently employed because of its high spatial and contrast resolution as well as its 3D imaging ability; in many cases CT is preferred based on the localisation of the nodule or other patient-related factors.

## Clinical indication

**Fig. 1:**
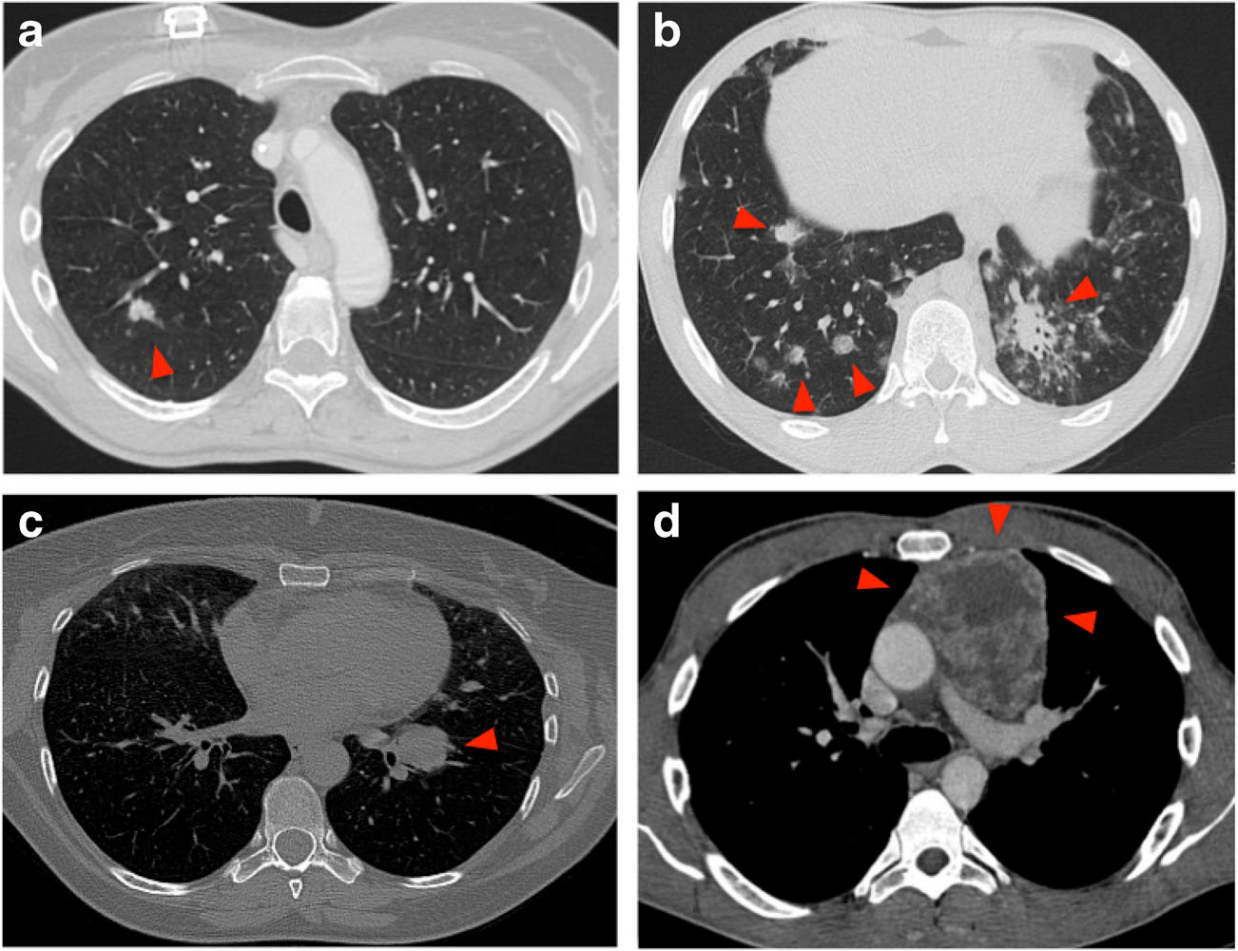
Indications for CT-guided chest biopsy. (a) Solitary pulmonary nodule. (b) Parenchymal infiltrates in which an infectious organism cannot be isolated. (c) Hilar mass following negative bronchoscopy. (d) Undiagnosed mediastinal mass

Clinical indications of imaging-guided chest biopsy have significantly changed from the introduction of the technique as a result of technical advances in needle technology, imaging modalities, pathological analysis and immunohistochemistry techniques. The introduction of PET-CT further modified these indications, reducing the percentage of unnecessary biopsies [2]. At present, the growing list of indications (Table 1) includes histological diagnosis of undetermined and otherwise not characterisable pulmonary, mediastinal and chest wall lesions as well as biopsy or re-biopsy of known malignant lesions to obtain histological material for targeted therapy. (Fig. 1).

**Table 1:** Indications for imaging-guided chest biopsy

Indications for imaging-guided chest biopsy
1. A new or enlarging solitary nodule or mass
2. Multiple nodules in a patient without known neoplastic disease or in prolonged remission
3. Focal parechymal infiltrates in which an infectious organism cannot be isolated
4. Diagnosis of hilar masses following negative broncoscopy
5. Undiagnosed mediastinal mass.
6. Bipopsy or re-biopsy of malignancy for targeted therapy.

### A new or enlarging solitary nodule or mass

The increasing clinical use of chest CT has resulted in an increasing incidental detection of small pulmonary nodules. In particular, pulmonary nodules are incidentally detected in around 8.5% of the general population [3] and in up to 51% of subjects with risk factors [4]. Therefore, a new or enlarging solitary nodule is the most common indication for imaging-guided chest biopsy. If a nodule is initially identified at conventional chest radiography, CT investigation is necessary to characterise the lesion, estimate the likelihood of malignancy [5] and identify lymphadenopathies or other accessible sites for biopsy (such as extra-thoracic metastases) [6, 7]. PET-CT examination may also be helpful in the management of pulmonary nodules larger than 1 cm, reducing the need of puncture of non-enhancing solid nodules [4–8] and with a sensitivity of 94% and a specificity of 83% for solid pulmonary nodules between 1 and 3 cm in diameter [9]. It must be taken in consideration that active inflammation (i.e. active tuberculosis, histoplasmosis, rheumatoid nodules) may cause false positives on PET-CT scans because of their high glucose metabolism [10]. On the other hand, low-grade malignant tumours, such as carcinoid [11] or low-grade adenocarcinoma [12], may produce false-negative results because of their low glucose metabolism. In these cases, careful follow-up with CT must be performed according to well-established guidelines to demonstrate regression/disappearance of the nodule after therapy or to address patients to biopsy if persistence or increase in size are evident [13].

### Multiple nodules in a patient without known neoplastic disease or in prolonged remission

Several benign conditions can appear with slowly enlarging or multiple new pulmonary nodules, such as rheumatoid arthritis, sarcoidosis or infectious diseases, including tuberculosis and fungal infections, particularly in immunosuppressed patients [14–16]. In these cases appropriate clinical work-up and CT follow-up are sufficient to exclude malignancy. Nevertheless multiple nodular lesions may be the initial metastatic presentation in a patient with an unknown primary tumour or the sign of disease progression in a patient with known primary tumour in prolonged remission. In these cases imaging-guided biopsy may be necessary to confirm or exclude malignancy.

### Focal parenchymal infiltrates in which an infectious organism cannot be isolated

When infectious organisms cannot be isolated from the culture of sputum, blood or lung lavage, lung biopsy can be necessary to obtain tissue samples for pathological examination. The biopsy sample can be cultured for diagnostic purposes to establish the most appropriate pharmacological treatment. Imaging-guided biopsy was demonstrated to have a sensitivity of 80% and a positive predictive value of 100% [17, 18].

### Diagnosis of hilar masses following negative bronchoscopy

Transbronchial needle aspiration during bronchoscopy or under ultrasound guidance is still considered the reference technique for the diagnosis of hilar masses [19, 20]. However, when bronchoscopy cannot be performed or is inconclusive, hilar masses can be accurately diagnosed by imaging-guided biopsy.

### Undiagnosed mediastinal mass

Mediastinal masses are most frequently located in the anterior mediastinum and include a variety of different entities, such as thymic malignancy, lymphomas, endocrine tumours and malignant germ cell tumours [21]. In the absence of typical clinical and imaging features, histological diagnosis is necessary [22]; imaging-guided biopsy should be always attempted in accessible lesions before diagnostic mediastinoscopy or in case of inconclusive results of mediastinoscopy.

### Biopsy or re-biopsy of malignancy for targeted therapy

Advanced non-small-cell lung cancer in progression after first-line therapy may need imaging-guided re-biopsy to detect a possible new biological profile and to guide therapeutic decision-making in more than one-third of cases. [23].

## Contraindications

Because of moderate risk of bleeding, abnormal clotting function or thrombocytopenia should be recognised and corrected before the procedure. Thus, oral anticoagulants and antiaggregant drugs should be reduced or stopped to reach an international normalised ratio (INR) of less than 1.5 and an activated partial thromboplastin time (aPTT) not more than 1.5 times the control value; platelet transfusion is recommended for counts <50,000/μl (Table 2) [24]. Suspected hydatid cyst or arterio-venous malformation should not be biopsied but can be identified on CT. Mechanical ventilation, which may lead to pneumothorax and favours air embolism, inability of a patient to co-operate during the procedure or to suspend respiration on request or control cough are the major contraindications. A unique functional lung is often considered another contraindication, while severe chronic obstructive pulmonary disease, pulmonary hypertension or cardiac insufficiency do not constitute absolute contraindications but increase the complication rate. [25].

**Table 2:** Abnormal values that should be corrected before the procedure

Patient management before procedures with moderate risk of bleeding	
INR	Correct to <1.5
aPTT	Should be corrected for values >1.5 × control
Plateles	Transfusion recommended for count <50.000/μl

## Imaging guidance techniques

**Fig. 2:**
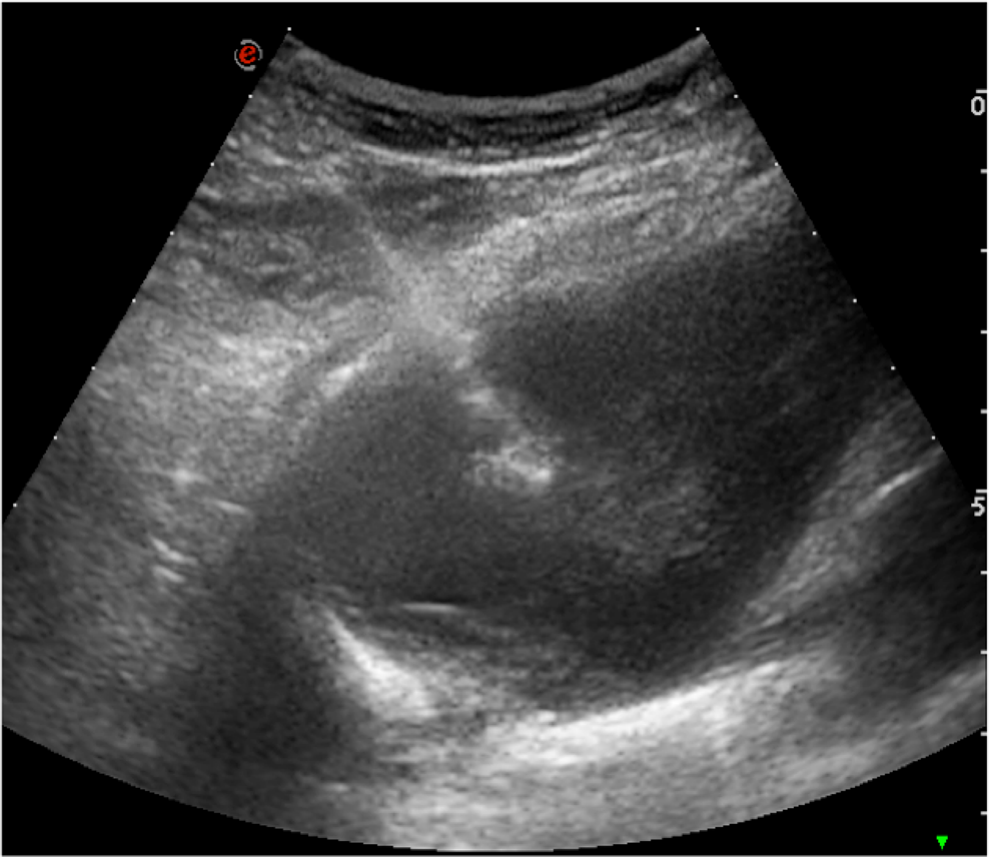
Ultrasound-guided chest biopsy of a pulmonary nodule in the right lower lobe

**Fig. 3:**
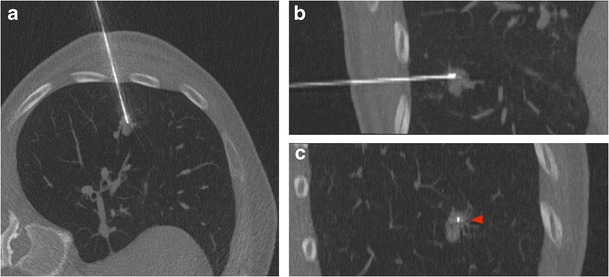
CT-guided chest biopsy of a pulmonary nodule in the left lower lobe. (a) Axial, (b) coronal and (c) sagittal intraprocedural CT scans showing the needle tip in the lesion (arrow)

**Fig. 4:**
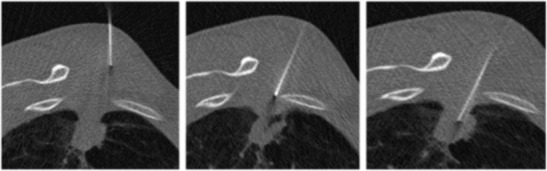
Axial images during fluoroscopy-guided chest biopsy of a solitary subpleural nodule

**Fig. 5:**
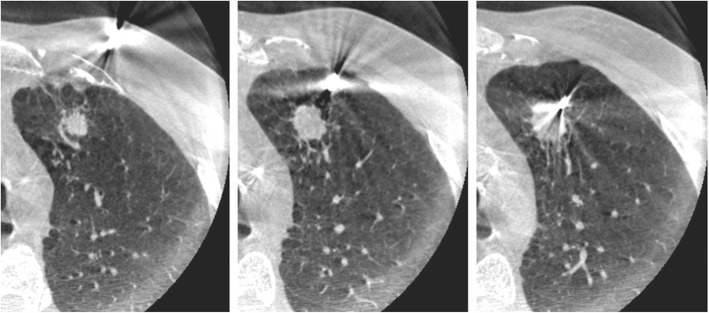
Progressive images of Cone-beam-guided chest biopsy of a pulmonary nodule in the left upper lobe

CT, fluoroscopy and US may all be used to guide chest biopsies and operators should be familiar with the advantage and limitations of each one [26]. Parameters affecting the selection of the most appropriate imaging technique are lesions site, size and visibility as well as its relationship with critical anatomical structures to be avoided [5]. Whenever possible, chest biopsies should be performed under US guidance (Fig. 2) to exploit the advantages of real-time monitoring without radiation exposure to patients and operators [27]; however, US guidance is limited to superficial lesions adjacent to the chest wall and/or to lesions delineated by pleural effusion sufficient to create a suitable interface for ultrasound penetration. In the vast majority of cases, CT (or cone-beam CT) (Figs. 3 and 4) is the preferred guidance method for chest biopsies due to its optimal spatial and contrast resolution as well as its 3D imaging ability. Furthermore an intravenous contrast agent can be used to differentiate target lesions from atelectasis, necrosis and vascular structures. CT-guided chest biopsies can be performed either with the step-and-shoot approach or with CT-fluoroscopy (Fig. 5): while the step-and-shoot approach allows 3D multiplanar reconstruction and significantly reduces the radiation dose to the patient and operators, it does not allow real-time monitoring of lesion movement during respiration and is strongly influenced by the patient compliance. On the other hand, real-time monitoring of lesions movement with CT-fluoroscopy guidance allows reducing needle passes and procedure time; however, the radiation dose (to both the patients and operator) is significant [28]. To simplify the approach and to increase the speed and reliability of step-and-shoot CT-guided chest biopsies, several technical advances have been proposed, including augmented reality and use of robotic platforms [29].

Furthermore, if a PET/CT is available, the needle should be led to the most enhancing area of the lesion to increase diagnostic accuracy.

## Biopsy procedure

### Patient positioning and instruction

The patient should be positioned prone, supine or lying on the side, based on the previously planned access site and needle trajectory. When needed, arms can be raised above the head to widen the intercostal spaces and obtain easier access. The procedure should be adequately explained to the patients with emphasis on the potential stinging sensation during the pleural puncture and breath-hold phases.

### Access site

**Fig. 6:**
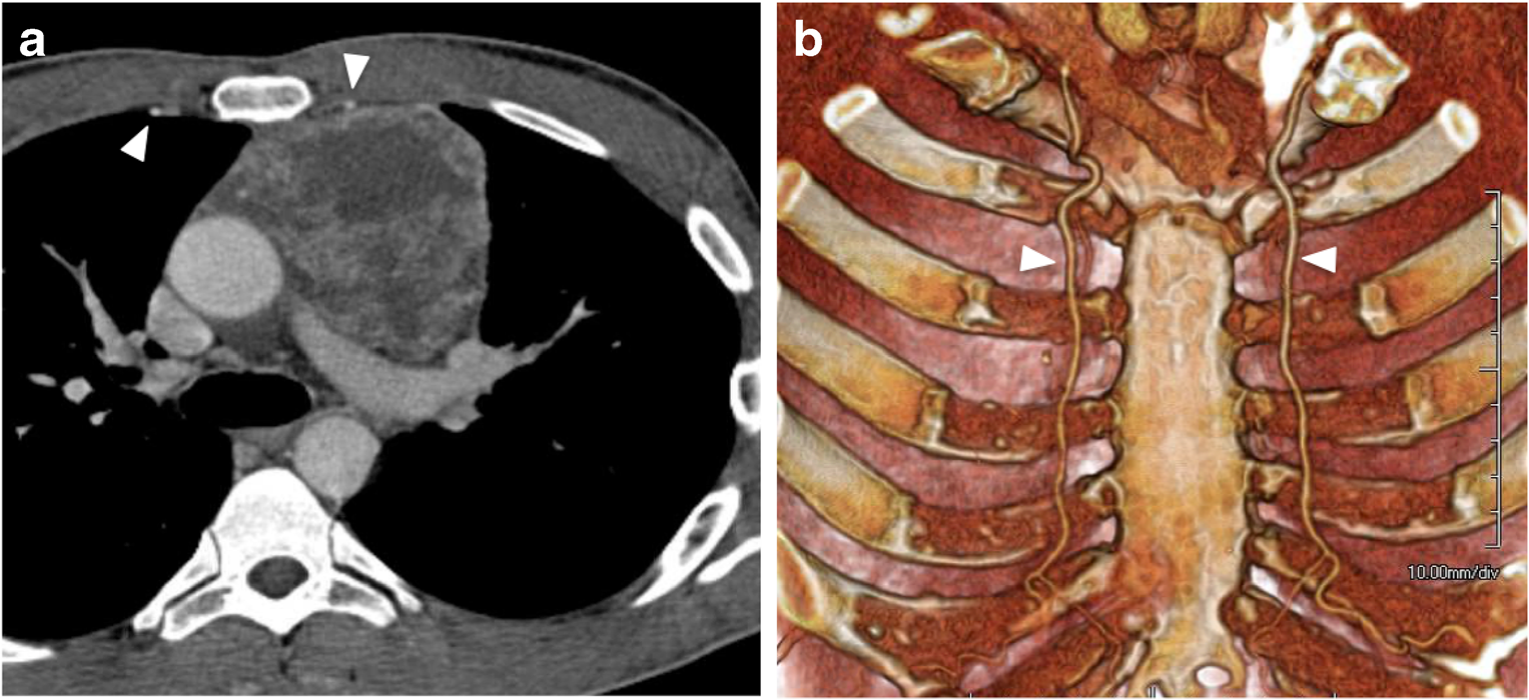
(a) Axial CT image and (b) 3D reconstruction showing internal mammary arteries (arrows), which must be avoided during the procedure

Whenever possible, the needle access site should be cephalic to the ribs to avoid intercostal vessel and nerves puncture (Fig. 6) [30]. In case of anterior parasternal access, internal mammary vessels should be carefully avoided [31]. Costal cartilage may traversed if needed but this may result in reduced needle mobility. The skin in the access site should be sterilised with standardised antiseptic solution and cutaneous and subcutaneous tissues should be infiltrated with lidocaine (lignocaine) up to a maximum dose of 20 ml of a 2% solution.

### Breath-hold techniques and needle manipulation

The breath-hold technique stabilises the positions of the diaphragm, pleural planes, lung, fissures and, ultimately, target lesions; however, breath-hold capabilities can be extremely different from patient to patient, and even the same subject may not be able to reproduce breath-hold during the procedure because of stress or fatigue. Therefore, it can be easier to target larger tumours (>2/3 cm) instructing the patient to breath freely with shallow respiration; even though the target lesions moves slightly, its large size may be sufficient to require few adjustments. In the remaining cases, the patient can be instructed to maintain an inspiratory or expiratory apnoea to allow easier access to target lesions.

### Needle types

Needle choice is based on the size and location of the lesion, intended needle trajectory, information expected from the pathologic sample, status of the lung parenchyma and operator preference. Some of the more commonly used fine-needle aspiration (FNA) devices include Chiba, Franseen, Westcott, MaxiCELL, Greene (Cook) and Turner (Cook) needles, which have circumferentially sharpened tips allowing for sampling. Core biopsy needles are designed to collect a small piece of tissue intended for surgical pathology analysis and can be designed as end-cutting or side-cutting devices. The most commonly used core biopsy needle is the Tru-Cut, which consists of an outer cutting cannula and an inner slotted stylet. The Temno core biopsy device is another similar commonly used automatic core biopsy needle. The Biopince full core is an automated end-cutting needle that produces a full cylindrical core specimen. The decision to perform core biopsy or both core biopsy and FNA is multifactorial and highly institution-/operator-dependent [28]. The number of passes needed per procedure has not been defined, but the decision to perform more than one puncture depends on procedure difficulty, risk of complications, quality of the first specimen obtained and the pathologist’s requests. The presence of an on-site pathologist may reduce the number of biopsies needed [5]. Also the use of a coaxial, a larger-bore needle that is kept in place to maintain access to the target lesion, may allow multiple tissue sampling, reducing repeated pleural or soft tissue punctures [32]. Several techniques have been proposed to seal the path of the needle after its removal to reduce the risk of pneumothorax and haemorrhage; the most successful option is to create a blood patch with autologous venous blood [33, 34].

## Diagnostic accuracy

### Ultrasound versus CT

In a recent meta-analysis, Di Bardino et al. compared the diagnostic accuracy of ultrasound and CT-guided thoracic biopsy. The overall pooled diagnostic accuracy of ultrasound-guided biopsy was 88.7% (446/503), with a sensitivity of 91.5% (366/400) and a specificity of around 100% for the diagnosis of malignancy. The overall pooled diagnostic accuracy of CT-guided biopsy was 92.1% (9567/10,383), with a sensitivity of 92.1% (7343/7975) and a specificity of around 100% for the diagnosis of malignancy (Tables 3 and 4) [35]. These data show a relative superiority of CT as guidance method, but data on CT-guided biopsies were more significant because of the higher number of cases in comparison with US-guided biopsies.

**Table 3:** Test characteristics for different imaging guidance techniques (ultrasound vs. CT), biopsy techniques (FNAB vs. core biopsy) and lesion location (pamediastinal vs. peripheral)

		Accuracy	Sensitivity	Specificity
Imaging guidance techniqueE	Ultrasound-guide biopsy	88.7%	91.5%	100%
	CT-guide biopsy	92.1%	92.1%	100%
Biopsy technique	FNAB	64–97%	82–99%	86–100%
	Core biopsy	93%	89%	97%
Lesion location	Paramediastinal	95.4%	95.6%	100%
	Peripheral	94.7%	94.2%	100%

**Table 4:** Safety profile of imaging-guided biopsy

	PTX	Haemorrhage
CT-guided biopsy	20.5%	2.8%
Ultrasound-guided biopsy	4.4%	–
Fluoroscopy-guided biopsy	11.1%	–

### FNAB versus core biopsy

There is a wide variation in reporting diagnostic accuracies of fine-needle aspiration biopsy (FNAB) between different institutions, ranging from 64% to 97%. The false-negative rate of FNAB varies, occurring in 6 to 54% of biopsies. The sensitivity, specificity and accuracy of FNAB for pulmonary lesions are 82 to 99%, 86 to 100% and 64 to 97%, respectively [28]. Core biopsy has been shown to have slightly higher overall sensitivity, specificity and accuracy, with respective values of 89%, 97% and 93% (Table 3). Several recent papers advocate the use of 18- and 20-gauge cutting needles as well as coaxial techniques to improve the diagnostic yield, reporting diagnostic accuracies of 74–95% for the diagnosis of malignancy. Charig and Phillips reported similar accuracy of FNAB and core biopsy when an on-site pathologist was available; in a similar setting Capalbo et al. observed a greater diagnostic accuracy of FNAB compared to core needle biopsy (94.83% vs. 81.82. %) [36]. Nowadays, the test of genetic mutations is important to plan targeted therapies in patient with lung cancers. With regard to this point, Schneider et al. observed a statistically significantly higher number of samples sufficient for molecular testing in core biopsy than FNAB (67% vs. 46%; *P* = 0.007) [37].

### Central versus peripheral lesions

Various studies evaluated the effects of lesion location on the diagnostic accuracy of imaging-guided biopsy in chest tumours. In a paper from Layfield et al. [38], the sensitivity in diagnosing lung tumours decreased from 100% in peripheral lesions to 82% in central lesions; however, this study was conducted in 1996 with basic CT equipment, as demonstrated by the striking differences in sensitivity between fluoroscopy guidance (97%) and CT guidance (80%). Yamagamy et al. [39] demonstrated that some peripherally located lesions are not accessible at all with conventional CT guidance because of their relationship with rib arcs, thus requiring needle positioning under CT-fluoroscopy guidance. Finally, Wang et al. [40] recently compared the rates of complications and diagnostic accuracy of CT-guided biopsy in peripheral versus paramediastinal lesions, demonstrating how this technique may be safe and reliable even for deeply located tumours; in particular, their paper reports diagnostic accuracy of 95.4% in paramediastinal lesions and 94.7% in peripheral lesions, with a sensitivity of 95.6% and 94.2% respectively (Table 3).

## Post-procedural care and complications

Once the biopsy has been performed, a CT scan of the chest is obtained to identify any immediate post-procedural complications. According to some authors, the patients should be rolled over onto the punctured side to reduce the risk of delayed PNX; anyway, this is a controversial opinion, since other authors have reported no benefits of putting patients in the “biopsy down position” [41]. Following measures include observation and monitoring of vital signs for at least 4 h. Chest films are usually acquired after 4 h to detect possible asymptomatic PNX. If the clinical suspicion of a PNX arises, chest radiography must be obtained immediately. In low-risk patients, many interventional radiology services reasonably perform lung biopsies on an outpatient basis, with discharge at 4 h and readmission only if symptoms develop [42].

### Pneumothorax

**Fig. 7:**
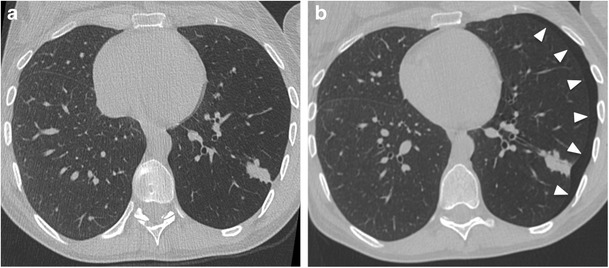
CT-guided chest biopsy of solitary pulmonary nodule in the left lower lobe. (a) Axial CT image before the procedure showing the subpleural nodule. (b) Post-procedural axial CT image showing small PNX (arrows) as a complication of transthoracic biopsy

PNX is the most common complication after imaging-guided chest biopsy; it is most frequently detected after lung biopsy but can also occur even after biopsies of mediastinal, pleural and chest wall lesions. Usually PNX occurs during or immediately after the procedure and it is detected on post-procedural control scans. The incidence of PNX has been reported to be up to 61% with an average risk of 20% [43–46]. Risk factors for PNX can be related to patient or lesions features, but also to the biopsy technique. In particular, the rate of PNX increases with the patient age and severity of underlying lung disease (e.g. emphysema or chronic obstructive disease) [47, 48] as well as in smaller and deeper lesions [49, 50]. Technical risk factors include the type and size of biopsy needle, longer procedure duration, biopsies in the middle or lower lobe, transgression of a fissure and multiple needle repositioning or pleural passes [51]. A PNX developed during the procedure can be immediately aspirated through the introducer needle or a separate needle inserted into the pleural space, preventing further enlargement; however some authors suggest placing a chest tube if aspirated air is greater than 670 ml [40]. PNXs developed after the procedure (Fig. 7) are often small and asymptomatic and can be managed conservatively by monitoring vital signs and performing serial chest films (at 1 and 4 h) [45, 52]. Nevertheless, in a minority of cases (1–14%), PNX can be significant (>30% of lung volume), increase over time or become symptomatic. In these cases small-calibre, 6- to 9-French catheters can be safely and easily placed under CT guidance [41, 53, 54].

### Pulmonary haemorrhage and haemoptysis

**Fig. 8:**
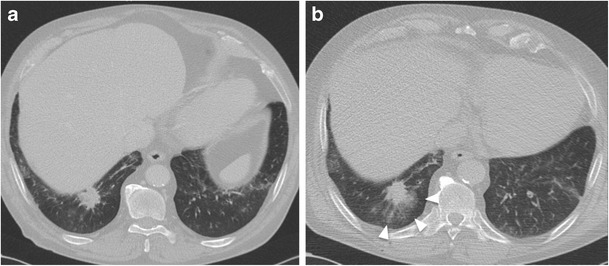
CT-guided chest biopsy of a pulmonary nodule. (a) Axial CT image before the procedure showing a pulmonary nodule in the right lower lobe. (b) Post-procedural axial CT image demonstrates perilesional haemorrhage as ground-glass opacity around the nodule (arrows)

Pulmonary haemorrhage represents the second most common complication after imaging-guided biopsy. PH may occur with or without haemoptysis and can be easily detected on screening post-biopsy CT scan as a perilesional or needle tract ground-glass opacity (Fig. 8). The occurrence rates of PH are estimated to be from 4 to 27% (with an average incidence of 11%), while haemoptysis risk is up to 5% [55, 56]. Usually this complication does not need any treatment and the only recommendations is to place the patient in a lateral position, with the biopsy side down, to avoid aspiration of blood into the unaffected lung [44]. Occasionally a larger, higher-grade PH occurs and oxygen as well as pro-coagulative therapy may be needed. Risk factors for higher-grade PH include older age, female sex, emphysema, pulmonary hypertension, coaxial technique, subsolid lesions, nonsubpleural location and lesion size smaller than 3 cm [57, 58]. Avoiding PH is important to manage patients with abnormal coagulation profiles and to correct the diathesis before the procedure. In such patients, more invasive biopsy techniques that imply the use of the core needle and coaxial technique should be avoided as should prolonged procedures with extended needle paths [45, 59]. Finally, haemothorax is an extremely rare and more severe complication, usually due to puncture of an intercostal or less commonly a large thoracic vessel, or mammary vessels in the case of an anterior parasternal biopsy.

### Air embolism

**Fig. 9:**
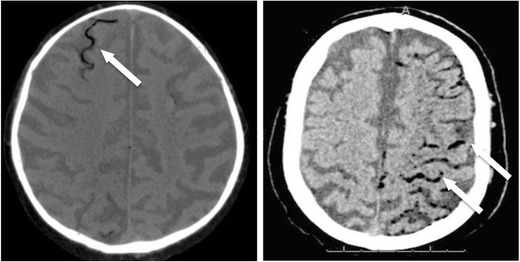
Post-procedural CT scan after chest biopsy showing two different patients with air embolism in cerebral vessels (arrows)

The occurrence of systemic air embolism (SAE) in the left atrium, left ventricle or systemic circulation is a rare (incidences between 0.01% and 0.21%), but potentially fatal (by brain or cardiac infarct) event [59] (Fig. 9). There are three mechanisms in particular responsible for SAE during biopsy: placement of the needle tip in a pulmonary vein, formation of a bronchial-venous or alveolar-venous fistula and opening the outer cannula of a coaxial biopsy needle to the atmosphere. Risk factors can be biopsy of cystic or cavitary lesions (i.e. vasculitic granulomas), coughing during the biopsy and positive pressure ventilation [60]. In a study from Freund et al., asymptomatic SAE detectable at CT after lung biopsy was reported to be as high as 3.8% (23/610 patients), whereas the symptomatic cases were 0.49%. In the meta-analysis from Tomiyama et al. [61, 62], the incidence of SAE ranges from 0.001% to 0.003%, being influenced by the depth of needle penetration into the lesion, endotracheal anaesthesia, location of the lesion above the level of the left atrium and prone position of the patients [61].

### Tumour seeding

Tumour seeding through the needle tract represents a very rare complication with a prevalence reported in the literature between 0.012 and 0.061% [45]. The real clinical relevance is still discussed, but it is obvious that tumour seeding along the needle tract can significantly change patient management and life expectancy and should be strictly avoided [63]. Tumour seeding is reported to be more frequently observed after imaging-guided core needle biopsy of pleural mesothelioma [64].

### Complications by imaging guidance technique

In the meta-analysis from Di Bardino et al. US-guided biopsy was generally very well tolerated and safe, with a pooled incidence of PNX of 4.4% (22/503) in the reported papers. This looks favourable compared to CT-guided biopsy, but is also obviously correlated to a statistical bias in lesion position since the targets of US-guided biopsies are usually adjacent to or infiltrating the pleural surface, with a very low risk of PNX.

Compared with the conventional step-and-shoot approach for CT-guided biopsy, CT fluoroscopy is faster and requires fewer needle passes, resulting in a decrease of procedure duration and fewer complications. In particular Heck et al. [48] showed a trend toward a lower PNX rate when using CT fluoroscopy as guidance method. Similar results have been obtained by Kim et al. [65], with a decrease from 27.1% to 11.1% in PNX rates when using CT fluoroscopy.

## Conclusions

In conclusion, imaging-guided chest biopsy is an interventional procedure of pivotal importance for several clinical conditions of pneumological, oncological and surgical interest. This procedure may appear very simple and linear, but radiologists approaching it for the first time must consider several clinical and technical variables significantly affecting the final results, in terms of both diagnostic accuracy and patients’ safety.
